# Supplementary material for: Healthcare financing and social protection policies for migrant workers in Malaysia
Source: PLoS One. 2020 Dec 9;15(12):e0243629. doi: 10.1371/journal.pone.0243629 (PMC7725341; doi:10.1371/journal.pone.0243629)
Supplement: S1 Table — (DOCX) [file pone.0243629.s002.docx]

#### S1 Table. Charges for Malaysian citizens and non-citizens at public clinics and hospitals.

|  | **MALAYSIAN CITIZEN** | | **NON- CITIZEN^1,2^** | | |
| --- | --- | --- | --- | --- | --- |
| **Ward Deposit** | **Medical** | **Surgical** | **Medical** | **Surgical** | **Obstetrics and Gynaecology** |
| 1st Class | RM 700 (USD 173) | RM 1,100 (USD 273) | RM 7,000 (USD 1735) | RM 11,000 (USD 2726) | RM 7,000 (USD 1735) |
| 2nd Class | RM 200  (USD 50) | RM 400 (USD 99) | RM 3,000 (USD 99) | RM 5,000 (USD 1239) | RM 5,000 (USD 1239) |
| 3rd Class | RM 20  (USD 5) | RM 30 (USD 7) | RM 1,400 (USD 347) | RM 2,800 (USD 694) | RM 2,800 (USD 694) |
| **Daily Ward Charges** | **Air-conditioned** | **Non Air-conditioned** |  |  |  |
| 1st Class |  |  |  |  |  |
| 1 bedded room | RM 120 (USD 30) | RM 90 (USD 22) | RM 320 (USD 79) |  |  |
| 2 bedded room | RM 90 (USD 22) | RM 60 (USD 15) | RM 240 (USD 59) |  |  |
| 4 bedded room | RM 60 (USD 15) | RM 45 (USD 11) | RM 200 (USD 50) |  |  |
| 2nd Class | RM 40 (USD 10) | RM 25 (USD 6) | RM 180 (USD 45) |  |  |
| 3rd Class | RM 3 (USD 1) | RM 3 (USD 1) | RM 160 (USD 40) |  |  |
| **In-Patient Treatment Charges** |  |  |  |  |  |
| 1st Class | RM 15 (USD 4) | | RM 100 (USD 25) | |  |
| 2nd Class | RM 5(USD 1) | |  |  |  |
| 3rd Class | Free | |  | |  |
| **Out-Patient Treatment Charges** |  | |  |  |  |
| Out-Patient Department | RM 1 (USD 0.25) | | RM 40 (USD 10) | |  |
| Specialist Clinic | RM 5(USD 1.24) | | RM 120 (USD 30) | |  |

NOTE. All medical charges are reported in Malaysian Ringgit (RM) and United States Dollars (USD).

^1^ Treatment charges for non-citizens do not include investigation, procedure or medication

^2^ Exception is given to non-citizens with Permanent Residence status

Source: Official Website of Hospital Kuala Lumpur. Ministry of Health (11)
