# Supplementary material for: Healthcare financing and social protection policies for migrant workers in Malaysia
Source: PLoS One. 2020 Dec 9;15(12):e0243629. doi: 10.1371/journal.pone.0243629 (PMC7725341; doi:10.1371/journal.pone.0243629)
Supplement: S2 Table — (DOCX) [file pone.0243629.s003.docx]

#### S2 Table. Estimated annual levies collected for migrant workers by sector, 2019.

| **SECTOR** | **Workers (Peninsular)** | **Workers  (Sabah/ Sarawak)** | **Levy ^1^ (Peninsular)** | **Levy ^1^ (Sabah/ Sarawak)** | **Total Levy  (Malaysia)  (millions)** |
| --- | --- | --- | --- | --- | --- |
| Manufacturing | 659,925 | 39,505 | RM 1,850  (USD 458) | RM 1,010  (USD 250) | RM 1,261  (USD 313) |
| Construction | 410,665 | 27,599 | RM 1,850  (USD 458) | RM 1,010  (USD 250) | RM 788  (USD 195) |
| Plantation | 150,228 | 122,851 | RM 640  (USD 159) | RM 590  (USD 146) | RM 169  (USD 42) |
| Agriculture | 117,077 | 39,257 | RM 640  (USD 159) | RM 410  (USD 102) | RM 191  (USD 23) |
| Services | 289,421 | 16,731 | RM 1,850  (USD 458) | RM 1,490  (USD 369) | RM 560  (USD 139) |
| Domestic work | 118,403 | 10,765 | - | - | - |
| **TOTAL** | **1,745,719** | **256,708** |  | **RM 181**  **(USD 45)** | **RM 2,868**  **(USD 711)** |

^1^ Levies vary by employment sector and employment in Peninsular Malaysia or Sabah and Sarawak in East Malaysia.
All costs are reported in 2018 Malaysian Ringgit (RM) and United States Dollars (USD).
Source: [7, 47]
