# Supplementary material for: Healthcare financing and social protection policies for migrant workers in Malaysia
Source: PLoS One. 2020 Dec 9;15(12):e0243629. doi: 10.1371/journal.pone.0243629 (PMC7725341; doi:10.1371/journal.pone.0243629)
Supplement: Attachment — Submitted filename: PONE D 20 11263.pdf [file pone.0243629.s007.pdf]

Healthcare financing and social protection policies for migrant workers in Malaysia

Comments

Good manuscript, well written.

The following are my suggestions for improvement.
Line 62, 63: Need reference
Line 117: 37 in-depth interviews of 44 individuals: Need to elaborate the division of the 44 individuals to get the 37 in-depth interviews.

Line 172, and 346: Fig 1 and Fig 2. Is this a short form for figure? If yes it is not stated clearly. I cannot find fig 1 nor fig 2, what follow is more of a description

Line 233 and line 405: Qualitative 1 and Qualitative 1.

The presentation of results needs some organisation to make it easier to read and understand.
Example after the topic/heading i.e qualitative 1 (line233) it will be followed by sub-heading (line 234) this is not the case of line 307, there is no sub-heading.
