# Supplementary material for: Healthcare financing and social protection policies for migrant workers in Malaysia
Source: PLoS One. 2020 Dec 9;15(12):e0243629. doi: 10.1371/journal.pone.0243629 (PMC7725341; doi:10.1371/journal.pone.0243629)
Supplement: Attachment — Submitted filename: PLOS ONE Review.docx [file pone.0243629.s004.docx]

**Overall assessment:**

The article is timely in its contributions to the goals of UHC in an upper-middle-income country that attracts large numbers if migrant labor. It is well-written and the abstract is adequately summarized.

**Methodology:**

1. Largely qualitative with limited quantitative data; the approach is adequate to answer the objectives of the study.
2. Please clarify Table 1: the total number of participants is said to be 37 (Line 117) while the table gives a total of 44.

**Results**

1. Minor grammatical issues: line 180 and 181- Use ‘First’ instead of ‘Firstly’ and ‘Second’ instead of ‘Secondly’
2. Further explanation required on line 188. See side comments of main document
3. Correct ‘seven’ instead of 7
4. In general, the results section is detailed enough in terms its description of health policy evolution for migrant labor. However, I am reluctant to agree that the themes accurately address the aim of the study to “evaluate healthcare financing and social security policies for migrant workers; to identify policy gaps and opportunities for intervention” ....
5. …From my reading of the manuscript, the following key themes are evident and should be highlighted and adequately discussed with suggestion for way forward including health financing reforms:
   a. Weak implementation of health insurance and social protection policies

-this is evident in the lack of awareness of insurance scope by employer and employee; reluctance to report work-place injuries; a disempowered migrant labor-force that cannot assert itself to exercise its health insurance entitlements

-the authors need also to let readers understand why this (weakness in policy implementation) is happening.

b. Institutional discrimination of migrant workers

-evident in terms of differences in health services coverage between citizens and migrants; differences in compensation in cases of injury

-overall inequities in fees charged, OP and IP care at public facilities; etc.

c. Misalignment between services provided and migrant health needs: (Lines 253 – 4)

-this needs to be discussed in more details including examples and the reasons this is happening and the implications on migrant health

d. Mistrust in government initiatives by migrant workers (as highlighted in lines 467 – 477)

It will help the article a great deal if the authors could feature these major themes more prominently and discuss the sub-themes already in the article, including taking a closer look at their meaning for the goals of UHC in Malaysia (with examples from the interviews).

I’d encourage the authors to aggregate into major themes the sub-themes they have itemized on the article to give it the flow and concreteness it deserves because this article addresses the subject of migrant health which is a major source of inequity in health services.

A suggestion for further research would be a quantitative analysis of out-of-pocket spending by migrant workers; the scope of such spending; the drivers and implications on migrant worker well-being.
