# Supplementary material for: Healthcare financing and social protection policies for migrant workers in Malaysia
Source: PLoS One. 2020 Dec 9;15(12):e0243629. doi: 10.1371/journal.pone.0243629 (PMC7725341; doi:10.1371/journal.pone.0243629)
Supplement: Attachment — Submitted filename: Healthcare financing and social protection policies for migrant workers in Malaysia_aug 2020.docx [file pone.0243629.s006.docx]

**Healthcare financing and social protection policies for migrant workers in Malaysia**

The paper seeks to “examine the evolution of healthcare policy for migrant workers in Malaysia, while evaluating healthcare financing and social security policies and other options for fiscal space, in order to identify gaps and opportunities to improve migrant health financing and coverage.”

The paper is an important and interest read, researching an understudied population (migrant workers) and is of high policy relevance in Malaysia and beyond, in particular in other context with larger labour migrant populations. The analysis is supported by highly informative tables (in particular Table 3). The context is well-described for a reader who is not very familiar with the Malaysian context.

While this is of great interest, there are some important concerns.

**Major comments:**

- This analysis builds upon mixed methods (page 6), combining qualitative analysis (Policy document review, qualitative interviews) and quantitative data. While this is of interest, the abstract and methods section do not provide sufficient description of:
  - Why was this approach taken
  - Details about the different methods used
    - The policy literature review needs further detail. The authors describe: “For the document review, Malaysian health and labour laws, policy documents, guidelines and circulars relating to healthcare delivery to migrants, and reports of local and international organisations concerning migrant health were retrieved and analysed.”. The following questions remain: How were these identified? Which time frame (the results section write past two decades)? How were they analysed? In my view, it seems like a policy mapping more than review has been carried out (identification of policy and main content).
    - Quant. Component: “For the quantitative component, we analysed published data on medical revenues collected and outstanding revenues…” Please add additional information about how this information was collected (it sounds like data extraction more than data collection).
    - Related to these weaknesses, I do think the statement in the Discussion is an exaggeration “This policy analysis is unique as it combines qualitative interviews with document review and examination of the economic evidence, to examine the adequacy of available healthcare financing and social security schemes for migrant workers in Malaysia.” While the qual. analysis is indepth, it seems as the policy review is a policy mapping, and that the quant. data analysis seems to be data extraction/presentation of data. I am not convinced that the quant. data are quant. data, that are analysis as it seems that they are data presented to support information about the context more than analysis. The revised draft should be clearer about when primary data were collected/or analysed. I think it adds value, but should not be presented as quant. analysis unless this is done.
- Results
  - Reporting from qualitative interviews, findings are reported as “Those interviewed” or “interviewees” (page 17). It would be relevant to know more about who said this, given the heterogeneous sample.

**Minor comment:**

- Page 4, line 57-58:You refer to the Migration compact. While this is relevant, organisations such as ILO and IOM have been working to improve the rights of migrants for a longer period.
- Further discussion of Malaysian changes in health policies in relation to trends in the geographical region (or other labour-migrant dependent economies) would be of great interest.
