# Supplementary material for: Healthcare financing and social protection policies for migrant workers in Malaysia
Source: PLoS One. 2020 Dec 9;15(12):e0243629. doi: 10.1371/journal.pone.0243629 (PMC7725341; doi:10.1371/journal.pone.0243629)
Supplement: Attachment — Submitted filename: Response to Reviewers.docx [file pone.0243629.s009.docx]

We thank the previous three reviewers for taking the time to review and accept changes to the revised version of our manuscript. Their substantive comments and suggestions have strengthened the paper. We address the minor outstanding point below.

**Response to the Editor:**

The manuscript has been evaluated by four reviewers (three old, one new), and their comments are available below. I was a reviewer of the previous version the manuscript, and are now serving as a Guest Editor for the manuscript. The manuscript has improved substantially and three reviewers were satisfied with your responses and revisions. However, a new reviewer reiterate concerns raised regarding your claims of a mixed-methods study and triangulation is unclear. While you have added clarification on the policy analysis, we ask you to revise the manuscript to carefully address the methods concerns raised. A detailed description in the Study design section of the manuscript of why a mixed-method study design was chosen would be helpful, and reflection on its strengths and limitation in the Discussion section.

Use of the term ‘mixed methods analysis’ appears to be the sticking point. We omit use of this term in the revised Study design section as follows (Page 6, Line 93- 98):

***Study design***

*Policy document review and thematic analysis of qualitative interviews were combined with secondary quantitative data to evaluate healthcare financing and social security policies for migrant workers. We combined major themes from interview data with policy analysis and descriptive quantitative data is presented to contextualize findings, in an integrated Results section. Our mixed methods approach provides insights at critical moments in the analysis, rather than a standardized mixed methods research approach, which has been critiqued for taking an unreflective and mechanical approach to knowledge production[1].*

There are no current references to a mixed methods analysis in the Limitations section. As such, we see no need to make changes here.

**Response to Reviewer 5**

Reviewer #5: The previous reviewers questioned whether the way qualitative and quantitative work has been conducted and analyzed is consistent with the claim of a mixed-methods design. The authors' response is still unclear. Since there was no primary quantitative data collection and the methods employ no clear strategy (and clear description) e.g. triangulation to convincingly demonstrate validation of study questions using different methods, the mixed-methods claim is still unconvincing. Authors need to clarify this in methods and demonstrate this in results and discussion.

Use of the term ‘mixed methods analysis’ appears to be the sticking point. We omit use of this term in the revised Study design section, and clearly state that methods were combined in an integrated Results section. We specify that secondary quantitative data were descriptively analysed (Page 6, Line 93- 98):

***Study design***

*Policy document review and thematic analysis of qualitative interviews were combined with secondary quantitative data to evaluate healthcare financing and social security policies for migrant workers. We combined major themes from interview data with policy analysis and descriptive quantitative data is presented to contextualize findings, in an integrated Results section. Our mixed methods approach provides insights at critical moments in the analysis, rather than a standardized mixed methods research approach, which has been critiqued for taking an unreflective and mechanical approach to knowledge production[1]*

References

1.	Timans R, Wouters P, Heilbron J. Mixed methods research: what it is and what it could be. Theory and Society. 2019;48(2):193-216. doi: 10.1007/s11186-019-09345-5.
